# Structural and functional comparison of *Saccharomonospora azurea* strains in terms of primycin producing ability

**DOI:** 10.1007/s11274-020-02935-x

**Published:** 2020-09-29

**Authors:** Márk Kovács, Dénes Seffer, Ágota Pénzes-Hűvös, Ákos Juhász, Ildikó Kerepesi, Kitti Csepregi, Andrea Kovács-Valasek, Csaba Fekete

**Affiliations:** 1 PannonPharma Pharmaceutical Ltd., 7720 Pécsvárad, Hungary; 2 Faculty of Agricultural and Environmental Sciences, Institute of Biological Sciences, Szent István University, 2100 Gödöllő, Hungary; 3 Faculty of Sciences, Institute of Biology, University of Pécs, 7624 Pécs, Hungary

**Keywords:** Polyketide biosynthetic genes, Primycin, *Saccharomonospora azurea*, Structural and functional genomics

## Abstract

Emerging and re-emerging microbial pathogens, together with their rapid evolution and adaptation against antibiotics, highlight the importance not only of screening for new antimicrobial agents, but also for deepening knowledge about existing antibiotics. Primycin is a large 36-membered non-polyene macrolide lactone exclusively produced by *Saccharomonospora azurea*. This study provides information about strain dependent primycin production ability in conjunction with the structural, functional and comparative genomic examinations. Comparison of high- and low-primycin producer strains, transcriptomic analysis identified a total of 686 differentially expressed genes (DEGs), classified into diverse Cluster of Orthologous Groups. Among them, genes related to fatty acid synthesis, self-resistance, regulation of secondary metabolism and agmatinase encoding gene responsible for catalyze conversion between guanidino/amino forms of primycin were discussed. Based on in silico data mining methods, we were able to identify DEGs whose altered expression provide a good starting point for the optimization of fermentation processes, in order to perform targeted strain improvement and rational drug design.

**Electronic supplementary material:**

The online version of this article (10.1007/s11274-020-02935-x) contains supplementary material, which is available to authorized users.

## Introduction

Actinomycetales are known as natural producers of a wide range of biologically active secondary metabolites that exhibit remarkable clinical importance (Jakubiec-Krzesniak et al. 2018; Solecka et al. 2012). Primycin, produced by a Gram-positive (G+) filamentous bacteria *Saccharomonospora azurea,* is a 36-membered marginolactone antibiotic that possesses high antimicrobial activity against frequent Gram-positive pathogens, including clinically prevalent multidrug-resistant strains (Feiszt et al. 2014). In the last decade complete genome sequences have been obtained for several species of *Saccharomonospora* genus, however the available genomics information regarding biologically active metabolite production is still underrepresentated compared to other members of *Pseudonocardiaceae* family. Primycin was first described in 1954 to be produced by *Streptomyces primycini*, and thereafter by *Micromonospora galeriensis*, but none of these species was validly published (Szabó et al. 1976; Vályi-Nagy et al. 1954). In recent times, only *S. azurea* is known to has the ability to produce primycin (Csepregi et al. 2012; Klenk et al. 2012).

It is well documented that several clinically important bioactive secondary metabolites are synthesized on modular polyketide synthase (PKS) and non-ribosomal peptide synthase (NRPS) enzyme complexes via a conserved thiotemplate mechanism (Du and Lou 2010; Wang et al. 2014). Among them, macrolide antibiotics, represented by polyene and non-polyene compounds are one of the most effective antimicrobial agents. Primycin, like other guanidine-containing macrocyclic polyketides is also synthesized by the bacterial modular type I polyketide synthase pathway. In general, each PKS module minimally consists of a ketosynthase (KS), acyltransferase (AT), and an acyl carrier protein (ACP) catalytic domains, usually extended by ketoreductase (KR), dehydratase (DH), enolyreductase (ER) and thioesterase (TE) accessory domains. The 4-guanidinobutanoyl-COA starter unit of the primycin biosynthesis is derived from the three-step L-arginine precursor pathway, catalyzed by amine oxidase, amidohydrolase and acyl-CoA ligase enzymes (Hong et al. 2013). The activated 4-guanidinobutanoyl group is transferred to the loading ACP domain, performed by ACP S-malonyltransferase and subsequently the growing polyketide chain synthesis follows the canonical type I PKS biosynthetic logic. Finally, the TE domain catalyzes the release of the ACP-bound polyketide product through hydrolysis of the thioester bound. Although the modular genetic architecture of type I PKS is intensively reviewed (Hertweck 2009; Tsai and Ames 2009), knowledge about genetic factors controlling the yield and quality of secondary metabolites synthesis is limited.

The detailed genetic map of the extended primycin PKS gene cluster revealed the presence of glycosyltransferase and agmatinase encoding genes directly adjacent to the PKS. In the late step of primycin biosynthesis, transfer of the arabinosyl moiety onto the hydroxyl group of A1, A2 and A3 isoforms is catalysed by glycosyltransferase analogue to ECO-0501 biosynthesis pathway (Banskota et al. 2006). Agmatinase enzyme, as a member of the ureohydrolase superfamily, is responsible for catalyzing conversion between guanidino and amino forms of primycin, by a nucleophilic attack on the amidino carbon. The importance of agmatinase in primycin-like amino/guanidino marginolactones biosynthesis was highlighted by Hong and coworkers (Hong et al. 2016).

Even though a comparative structural genomic approach can provide important knowledge related to antibiotic gene clusters, it can not tell the whole story. To gain more insight into structure–function relationships beyond static analysis of biosynthetic gene cluster, the application of acombined approach of structural and functional genomics revealed several differentially expressed genes (DEGs), possibly involved in primycin biosynthetic pathway. Based on Cluster of Orthologous Groups (COG) and Gene Ontology (GO) systems, DEGs responsible for signal transduction, fatty acid synthesis and multidrug transport were emphasized.

## Materials and methods

### Bacterial strains and culture conditions

*S. azurea* DSM 44631, *S. viridis* DSM 43017, *S. glauca* DSM 43769 and *S. cyanea* DSM 44106 strains used in this study were purchased from Leibniz Institute, DSMZ-German Collection of Microorganisms and Cell Cultures, while *S. azurea* SZMC 14600 was originated from Szeged Microbiology Collection (SZMC). Bacterial cultures were stored as a suspension in Luria Bertani (LB) broth with 20% (v/v) glycerol at − 80 °C. Culture conditions were carried out according to Valasek et al. (2016). Briefly 1 mL bacterial cell suspension was inoculated into 50 mL seed medium containing 3% (w/v) soy flour, 4.2% (w/v) water soluble starch, 0.36% (w/v) NaCl, 0.6% (w/v) CaCO_3_, 0.5% (w/v) sunflower oil, pH 8.0, and incubated for 2 days at 37 °C in an orbital shaker at 200 rpm. Subsequently, 1 mL of accurately homogenized seed culture was transferred into 35 mL of fermentation medium containing 4% (w/v) soy flour, 4% (w/v) water soluble starch, 0.3% (w/v) NaCl, 0.5% (w/v) CaCO_3_, 0.3% (w/v) stearic acid, 0.1% (w/v) KH_2_PO_4_, 0.6% (w/v) sunflower oil (pH 9.5), and cultivated for 7 days at 28 °C in an orbital shaker at 200 rpm. *Bacillus subtilis* ATCC 6633 used for agar well diffusion assay was purchased from American Type Culture Collection (ATCC).

### Antimicrobial assay

Antimicrobial activity of n-butanol-ethanol-distilled water 1:1:2 (v/v) (BEW) extracts of 5 day fermented cells were determined by agar well diffusion assay (Balouiri et al. 2016). Accordingly, *Bacillus subtilis* ATCC 6633 test strain (approx. 10^6^ CFU/mL) was inoculated into liquid phase (50 °C) Mueller–Hinton agar (Biolab). After solidification of culture media, sample holding wells were prepared (9 mm diameter, 5 mm depth). Original extracts were diluted ten times with BEW, and a subsequently twofold dilution series was prepared from each sample in ethanol-phosphate buffer (1.16% (w/v) K_2_HPO_4_; 0.91% (w/v) KH_2_PO_4_; 25% (v/v) ethanol; 75% distilled water (v/v)). As a reference, standard crystallized primycin-sulphate stock solution (1 mg/mL in BEW) was diluted ten times in BEW, and subsequent dilution steps were performed in ethanol-phosphate buffer to get 100 µg/mL; 50 µg/mL; 25 µg/mL; 12.5 µg/mL; 6.25 µg/mL; 3.125 µg/mL; 1.56 µg/mL; 0.78 µg/mL; 0.39 µg/mL final concentrations of primycin. Each plate contained control wells for 100 µL known concentration of primycin-sulphate standard, as well as the same volume of sample extracts. Antibacterial activity was determined by the size of the inhibition zones after 20 h incubation at 30 °C. Primycin concentrations of extracts were calculated according to the calibration curve fitted trend line equation. Samples were obtained from three independent fermentations and triplicated as technical replicates.

### Chromatographic analysis

High performance liquid chromatography (HPLC) with diode array detection (DAD) and electrospray-mass spectrometry (ESI–MS) detection based analysis of *S. azurea* cell extracts were carried out according to Kovács et al. (2019). Primycin concentrations were determined from 0.5 mL fermentation media, collected between the third and seventh days of fermentation. All samples were prepared in three independent biological replicates and measured in technical triplicates. Statistical analysis was completed using one-way analyses of variance (ANOVA). Values are reported as mean ± SD (standard deviation) and results were expected statistically significant when p < 0.05.

### Comparative genomics tools

Genome sequencing methods of *S. azurea* SZMC 14600 have been described previously (Csepregi et al. 2012). Comparative genome analysis was carried out within the Integrated Microbial Genomes Database Expert Review (IMG-ER) system (https://img.jgi.doe.gov/cgi-bin/w/main.cgi) (Markowitz et al. 2009, 2012). Records of the annotated genomes are displayed by the following accession numbers in GeneBank: *S. azurea* SZMC 14600—AHBX01000000; *S. azurea* DSM44631—AGIU02000000; *S. viridis* DSM43017—ABUM01000000; *S. glauca* DSM43769—AGJI00000000; *S. cyanea* DSM44103—AHLY00000000. In silico DNA–DNA hybridization (DDH) values among *Saccharomonospora* species were calculated by using the Genome-To-Genome Distance Calculator (GGDC) web server (https://ggdc.dsmz.de) (Meier-Kolthoff et al. 2013). Distance values were determined by the recommended Formula 2 for incomplete draft genomes.

### Identification and in silico structural analysis of primycin PKS gene cluster

The primycin type I PKS gene cluster was identified and analyzed by antiSMASH (Antibiotics & Secondary Metabolite Analysis Shell) (Blin et al. 2019). Database searches for homologues genes and proteins were performed using the National Center for Biotechnology Information (NCBI) BLAST server (Altschul et al. 1990). Domain analysis and motif search were done by SMART (Simple Modular Architecture Research Tool) (Letunic et al. 2012), SBSPKS (Structure Based Sequence Analysis of Polyketide Synthases) (Anand et al. 2010) and MEME (Multiple Em for Motif Elicitation) (Bailey et al. 2009). Multiple sequences alignment were performed by CLUSTAL W (Larkin et al. 2007).

### Transcriptomic analysis

Total RNA was extracted from 50 mg of cell paste collected from fermentation medium after five days according to the method described by Stiekema et al. (1988). After DNase treatment (Promega RQ1-RNase-free DNase) RNA quantity was measured by Qubit 2.0 Fluorometer (Thermo Fisher Scientific), and quality was determined by Agilent Bioanalyzer 2100 instrument (Agilent RNA 6000 Nano reagent kit). High quality total RNA samples (RIN > 8.5) from pooled biological replicates were processed using the SOLiD total RNA-Seq Kit (Thermo Fisher Scientific) according to the manufacturer’s recommendation. Briefly 5 µg of pooled RNA was enriched by depleting ribosomal RNA using RiboMinus rRNA Removal Kit (Life Technologies), and the leftover was fragmented using RNaseIII. Following enzymatic fragmentation the 100–200 bp size fraction was selected and ligated with adaptors. The templates were reverse transcribed using ArrayScript Reverse Transcriptase. The cDNA library was purified using Qiagen MinElute PCR Purification Kit (Qiagen) and size-selected on a 6% TBE-Urea denaturing polyacrylamide gel. The 150–250 bp cDNA fraction was amplified using AmpliTaq polymerase, and purified by Agencourt AmPureXP Beads (Beckman Coulter). The concentration of each library was determined using the SOLiD Library TaqMan Quantitation Kit (Life Technologies), and clonally amplified on SOLiD P1 DNA Beads by emulsion PCR. Beads were deposited onto sequencing slides and sequenced on SOLiD V4 Instrument using 50-base sequencing chemistry.

### Bioinformatic analysis

RNA-Seq data was analyzed using Galaxy’s open source, web-based platform (https://usegalaxy.org) (Afgan et al. 2018). Reads of the whole transcriptome of S*. azurea* SZMC 14600 and *S. azurea* DSM 44631 were aligned to *S. azurea* SZMC 14600 genome using Bowtie2 alignment protocol. Transcriptome assembly and differential expression analysis were performed according to the Cufflinks RNA-Seq workflow (Trapnell et al. 2010). DEGs represented at least twofold change (> 2 or < − 2 in log_2_) were functionally annotated using Blast2GO version 5.2.4. software (Götz et al. 2008). To determine GO terms, functional annotation of each transcript was performed against the non-redundant (nr) protein database compiled by National Center for Biotechnology Information (NCBI) using BLASTx with 10^e−3^ e-value threshold. The set of DEGs were classified into COG categories based on the Joint Genome Institute (JGI) Integrated Microbial Genomes & Microbiomes (IGM/M) system.

### Real-time quantitative PCR analysis

The expression of the agmatinase encoding gene was measured by quantitative real-time PCR (qRT-PCR) by ABI Prism 7900 Sequence Detection System (Applied Biosystems). Total RNA obtained from three independent fermentation processes were isolated using Quick-RNA MiniPrep Kit (Zymo Research), and quantified by Qubit 2.0 Fluorometer (Thermo Fisher Scientific). Reverse transcription was performed by RevertAid Reverse Transcriptase (Thermo Fisher Scientific) applying 1 µg of total RNA according to the manufacturer’s instructions. Agmatinase encoding cDNA was amplified using gene specific primers (forward: 5′-GTTGAACAGATACCGCTCGTC-3′ and reverse: 5′-TGTCTCACTCCTGAAGACCTC-3′) and Ct values were detected by SYBR Green/ROX fluorescence chemistry (Thermo Fisher Scientific) in 25 µL final volume. The thermal profile was as follows: 95 °C for 10 min initial denaturation, followed by 40 cycles of 95 °C for 15 s, 60 °C for 30 s 72 °C for 30 s and a final dissociation step at 95 °C for 15 s, 60 °C for 15 s and 95 °C for 15 s. The relative gene expression was determined by using the ΔΔCt method, relative to glyceraldehyde 3-phosphate dehydrogenase (GAPDH) (forward: 5′-CTACACGCAGGACCAGAACC-3′ and reverse: 5′-GTTCAGTTCGGGCAGGACGA-3′) as an endogenous control. Each biological sample was measured in at least three technical replicates.

## Results

### Primycin producing ability

**Fig. 1:**
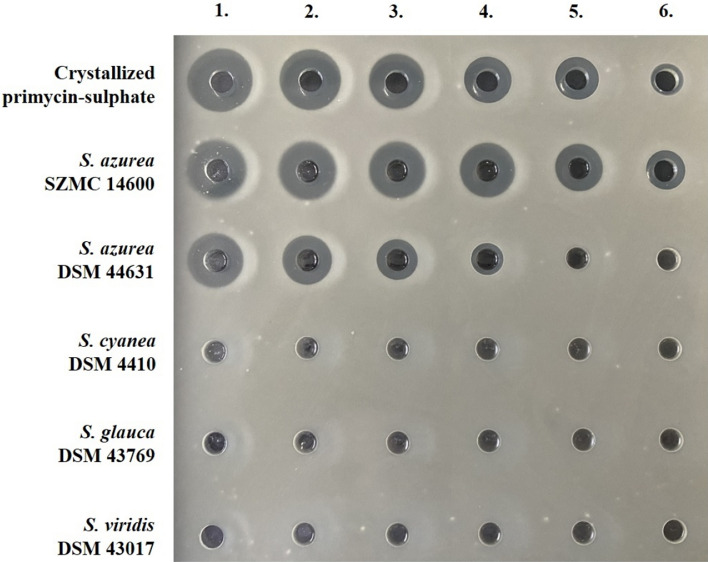
Agar well diffusion assay of primycin extracts from various *Saccharomonospora* species. Numbers from 1 to 6 indicate serial dilutions of fermented cells extracts. Wells of crystallized primycin-sulphate standard correspond to the following concentrations: (1) 12.5 µg/mL; (2) 6.25 µg/mL; (3) 3.125 µg/mL; (4) 1.56 µg/mL; (5) 0.78 µg/mL; (6) 0.39 µg/mL

**Fig. 2:**
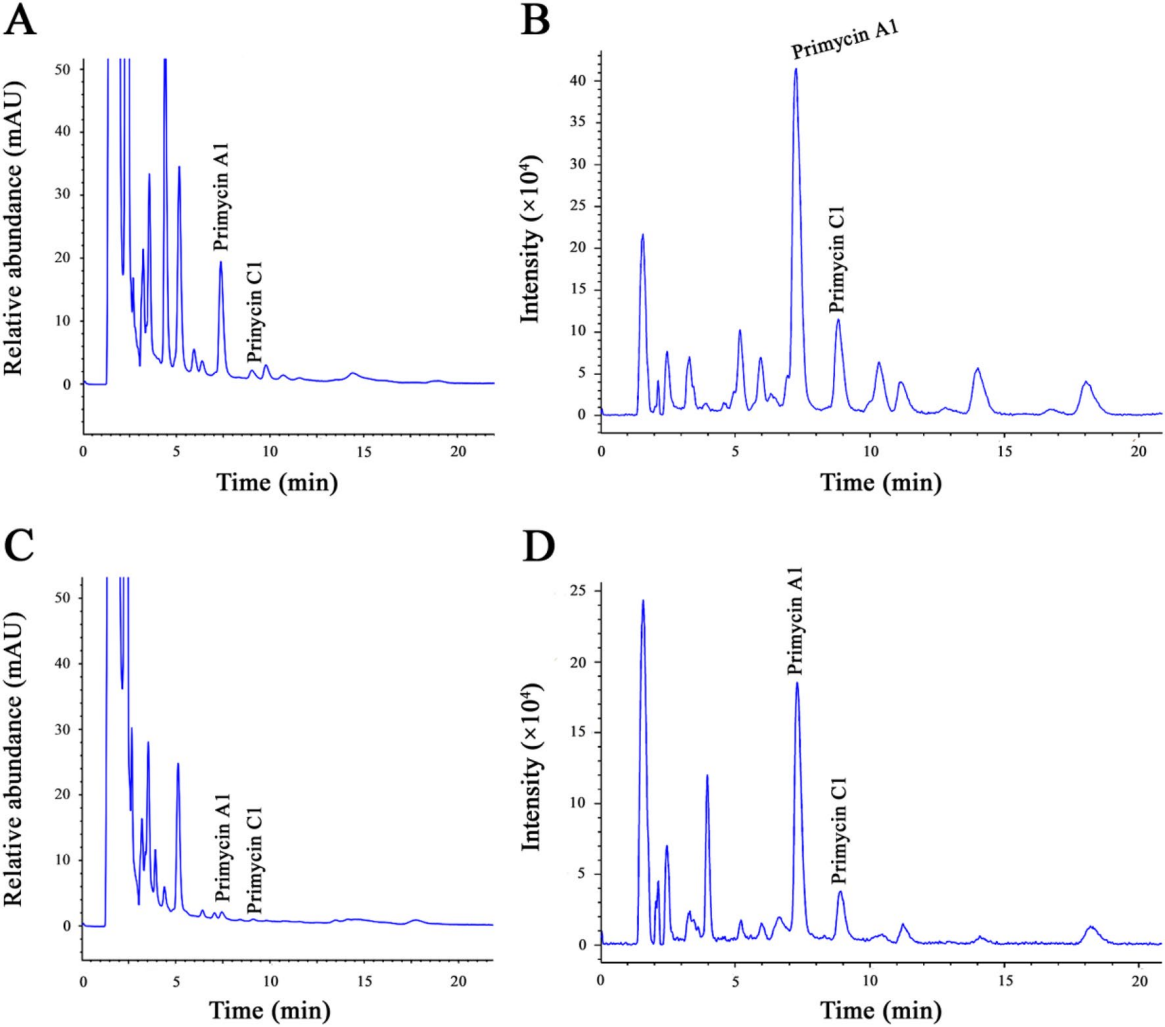
Reversed-phase high-performance liquid chromatography (HPLC) and electrospray-mass spectrometry (ESI–MS) chromatograms of the high-primycin producer *S. azurea* SZMC 14600 (**a**, **b**) and low-primycin producer *S. azurea* DSM 44631 (**c**, **d**) cells extracts. HPLC chromatogram (**a**, **c**) peaks with retention time of 7.38 min and 9.03 min correspond to primycin A1 (m/z 1078.7) and primycin C1 (m/z 946.5) respectively, based on molecular masses detected by ESI–MS (**b**, **d**). Data was acquired for 22 min over a 950 to 1150 m/z range in the positive ions mode

While *S. viridis* DSM 43017, *S. glauca* DSM 43769 and *S. cyanea* DSM 4410 strains can grow in the fermentation media, none of them were able to produce primycin, consequently no zone of inhibition was detected in the antimicrobial assay (Fig. 1). Each of the investigated *S. azurea* strains was primycin producer, nevertheless significant differences were observed in their production capacity. Based on agar well diffusion assay, calculated primycin concentration in case of *S. azurea* SZMC 14600 revealed approximately seven times more product (1173 ± 66.22 mg/L) compared to *S. azurea* DSM 44631 (168 ± 9.35 mg/L). Yields of primycin producing ability of *S. azurea* SZMC 14600 (high-producer) and DSM 44631 (low-producer) were confirmed by HPLC–DAD-ESI/MS analysis (Fig. 2) and summarized in Table 1.

**Table 1:** Primycin yields of *S. azurea* SZMC 14600 and *S. azurea* DSM 44631 strains measured by high performance liquid chromatography (HPLC) with diode array detection (DAD) and electrospray-mass spectrometry (ESI–MS) detection at different fermentation stages

Primycin concentration (mg/L)					
Strain	Day 3	Day 4	Day 5	Day 6	Day 7
*SZMC14600*	325.45 ± 6.18	728.24 ± 21.65	1113.89 ± 36.10	1248.08 ± 44.37	1261.06 ± 15.54
DSM 44631	190.36 ± 14.16	220.10 ± 8.68	197.66 ± 3.41	219.81 ± 6.45	210.48 ± 5.66

Concentration values (mg/L) are reported as means ± SD of three independent experiment (n = 3)

### Comparative structural genomics

**Fig. 3:**
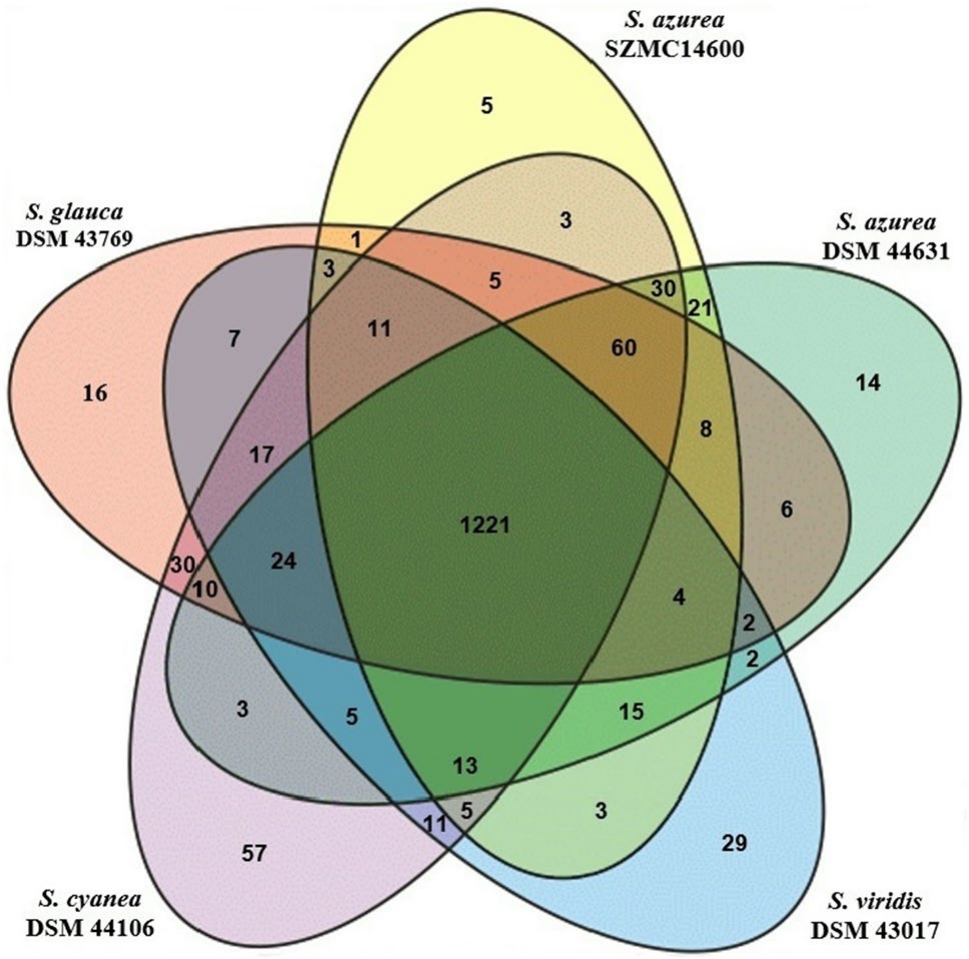
Venn diagram representation of the distribution of Clusters of Orthologous Groups (COGs) in the five analyzed *Saccharomonospora* genomes. Numbers are indicating shared and unique genes annotated in COG database

**Fig. 4:**
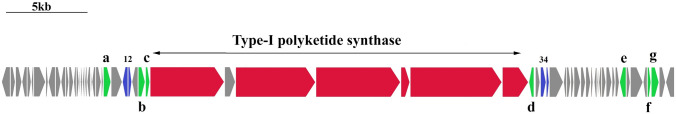
Schematic genetic map of the primycin biosynthetic gene cluster. Genes labelled by a, b, c, d, e, f and g and marked by green representing acyl-CoA carboxylase (EHK80154.1); glycosyltransferase (EHK80161.1); S-malonyltransferase (EHK80162.1); agmatinase (EHK80172.1); acyl-CoA ligase (EHK88415.1); amidohydrolase (EHK88411.1) and amine oxidase (EHK88410.1) respectively. Type-I polyketide synthase core genes are represented by red arrows. Numbers from 1 to 4 above blue colored arrows indicate genes encoding ABC transporter ATP-binding protein (EHK80158.1); ABC-transporter transmembrane protein (EHK80159.1); two-component histidine kinase (EHK80176.1) and two-component system response regulator (EHK80177.1) respectively

**Fig. 5:**
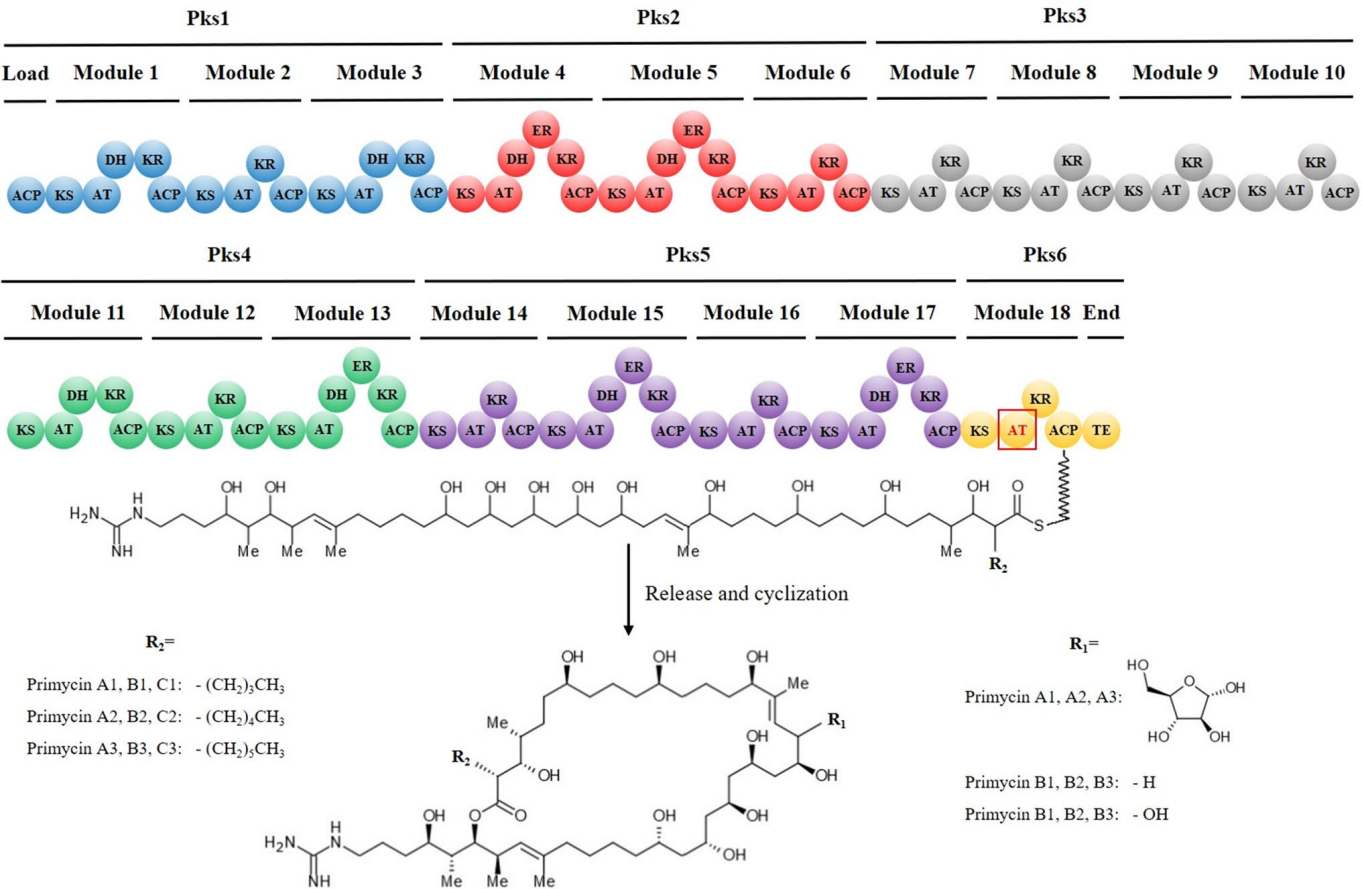
Module and domain organisations of primycin type-I polyketide synthase and predicted polyketide chain as well as the assembled final product in *Saccharomonospora azurea* SZMC 14600. AT domain responsible for unusual extender unit incorporation, located in module 18 is marked by red and boxed. The major forms of primycin complex determined by R_1_ and R_2_ side chains is shown at the bottom of the figure

Comparative whole genome analysis revealed a high percentage identity (93.4%) within the HSPs (high-scoring segment pairs) between *S. azurea* SZMC 14600 and *S. azurea* DSM 44631, while in case of other species identity in HSPs ranged from 28.0 to 20.3% (Table 1S). Based on overall similarities to antibiotic producer *S. azurea* strains, *S. cyanea*, *S. galuca* and *S. viridis* were selected for detailed in silico structural analysis. In order to further improve reliability of gene presence/absence polymorphism calling, the distribution of COG functional categories were determined. Protein characterized in COG represented on average 61.7% of total gene number (Table 2S). Comparative analysis of identified COG categories revealed 1221 proteins commonly present in all compared species. The graphical representation of COGs (Fig. 3) displays 40 *S. azurea* specific proteins which were not present in any additional member of the comparison. Among them 5 proteins were unique for *S. azurea* SZMC 14600, and 14 proteins were present only for *S. azurea* DSM 44631. Further genome mining efforts on previously reported high quality draft genome data of *S. azurea* SZMC 14600 revealed complete primycin biosynthetic gene cluster (PBGC) consisting of type I PKS core genes flanked by accessory genes (Fig. 4), however in case of *S. cyanea*, *S. galuca* and *S. viridis* the antiSMASH ‘in silico’ genome analysis revealed the absence of primycin biosynthetic gene cluster. The predicted primycin PKS model is composed of one loading and 18 separate extender modules, in good agreement with the chemical structure of primycin molecules (Fig. 5). The substrate specificity of PKS chain elongation is determined by AT domains. In case of primycin PKS, the amino acid sequence alignment of ATs revealed malonyl-CoA specific AT domains at module 4–10 and 12–16 having typical GHSx[LVIFAM]G and HAFH motifs (Fig. 1S). In modules 1–3, 11 and 17, methylmalonyl-CoA substrate specific motifs GHSx[QMI]G and YASH were observed (Fig. 2S) (Yadav et al. 2003; Zhang et al. 2019). Interestingly, antiSMASH in silicio analysis predicted unusual ethylmalonyl-CoA incorporation in module 18, however the presence of GHSQG and GAGH motifs define butylmalonyl-CoA, pentylmalonyl-CoA or hexylmalonyl-CoA incorporation (Fig. 3S).

### Transcriptomic analysis

To gained more insight into difference at gene expression level between high- and low-primycin producer *S. azurea* strains RNA-Seq has been applied. High throughput ligation based SOLiD V4 sequencing revealed 48 115 904 and 33 373 930 trimmed reads corresponding to 484 and 336-fold coverage of *S. azurea* SZMC 14600 and DSM 44631 genomes respectively. Following the Cufflinks RNA-Seq pipeline, transcriptomic analysis revealed 330 and 356 DEGs in a set of *S. azurea* SZMC 14600 vs. *S. azurea* DSM 44631 respectively at fold change cutoff of 2 in log_2_ scale. To identify functional categories of DEGs, GO enrichment analysis was performed. In case of high-primycin producer *S. azurea* SZMC 14600 strain, out of the 330 identified DEGs 253 were functionally annotated and classified into the three major GO categories as follows: 132 cellular component (C), 362 molecular function (F) and 231 biological process (P). Among cellular components, the most frequent categories were integral components of membrane (GO:0016021), cytoplasm (GO:0005737), plasma membrane (GO:0005886), ATP-binding cassette (ABC) transporter complex (GO:0043190), and intracellular (GO:0005622). Regarding molecular functions, ATP binding (GO:0005524), DNA binding (GO:0003677), metal ion binding (GO:0046872), hydrolase activity (GO:0016787), transferase activity (GO:0016740), and methyltransferase activity (GO:0008168) were the most numerous categories. The top five biological component categories included oxidation–reduction process (GO:0055114), transmembrane transport (GO:0055085), regulation of transcription, DNA-templated (GO:0006355), methylation (GO:0032259), and metabolic process (GO:0008152). In case of low-primycin producer strains, out of the 356 identified DEGs, 276 were functionally annotated and classified into: 133 cellular component (C), 332 molecular function (F) and 247 biological process (P). Representation of dominant GO categories in case of low-producer *S. azurea* DSM 44631 were practically identical (Fig. 4S).

**Fig. 6:**
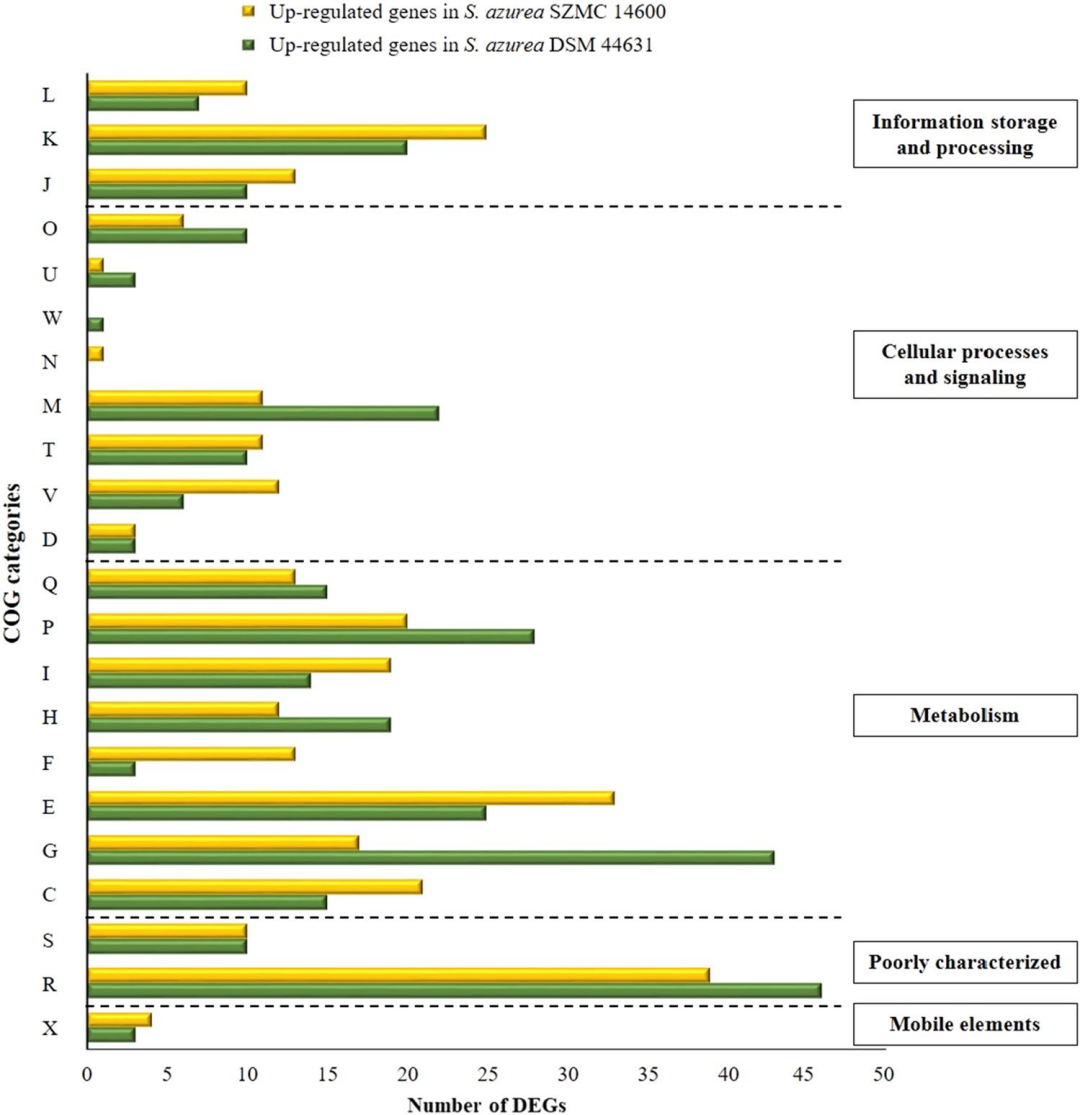
Representation of Clusters of Orthologous Groups (COGs) functional categories of differentially expressed genes (DEGs) in *S. azurea* SZMC 14600 and *S. azurea* DSM 44631. Abbreviations of COG functional categories: (C) Energy production and conversion; (D) Cell cycle control, cell division, chromosome partitioning; (E) Amino acid transport and metabolism; (F) Nucleotide transport and metabolism; (G) Carbohydrate transport and metabolism; (H) Coenzyme transport and metabolism; (I) Lipid transport and metabolism; (J) Translation, ribosomal structure and biogenesis; (K) Transcription; (L) Replication, recombination and repair; (M) Cell wall/membrane/envelope biogenesis; (N) Cell motility; (O) Post-translational modification, protein turnover and chaperones; (P) Inorganic ion transport and metabolism; (Q) Secondary metabolites biosynthesis, transport and catabolism; (R) General function prediction only; (X) Mobilome: prophages, transposons; (S) Function unknown; (T) Signal transduction mechanisms; (U) Intracellular trafficking, secretion and vesicular transport; (V) Defense mechanisms; (W) Extracellular structures

The possible function of DEGs were also predicted and classified by aligning to the COG database. COG assignment resulted 294 and 313 DEGs in comparison of high- and low-primycin producer respectively, corresponding to 22 specific categories. Gene products without related COGs, predominantly encoding hypothetical proteins, or assigned into general function prediction only (R), and function unknown (S) categories were filtered out. Among functional categories amino acid transport and metabolism (E), transcription (K) and energy production and conversion (C) were predominant in *S. azurea* SZMC 14600. Interestingly, analysis of the low-primycin producer *S. azurea* DSM 44631 strain revealed orthologous sequences involved in different biological processes such as carbohydrate transport and metabolism (G), inorganic ion transport and metabolism (P), furthermore notable differences were also observed in a number of assigned genes (equal to up- and down-regulation) as it is summarized in Table 3S. Taking into consideration the most characteristics expressional changes of high- and low-antibiotic producer strains, the following COG categories were remarkably down-regulated in SZMC 14600: carbohydrate transport and metabolism (G), cell wall/membrane/envelope biogenesis (M), inorganic ion transport and metabolism (P), and coenzyme transport and metabolism (H). In contrast, the majority of DEGs belonging to COGs involved in nucleotide transport and metabolism (F), amino acid transport and metabolism (E), energy production and conversion (C) and defense mechanisms (V) were up-regulated in the high-producer strain (Fig. 6). The analysis of the primycin PKS core biosynthetic genes with respect to expression levels revealed no differences among the high- and low-primycin producer *S. azurea* due to the two strains had comparable expression levels. As we presented in the schematic genetic map of the primycin gene cluster (Fig. 4), the agmatinase encoding gene (EHK80172.1) is located at the 3′ end of the PKS. According to the RNA-Seq analysis, the agmatinase encoding gene belonging to the amino acid transport and metabolism (E) COG category was up-regulated in the primycin overproducer with 2.62 log_2_ fold-change. The up-regulation tendency of agmatinase gene in *S. azurea* SZMC 14600 was confirmed by qRT-PCR (Fig. 5S).

It is well known that polyketide and fatty acid (FA) synthesis are evolutionary closely related processes, and the two megasynthase assembly lines use homologous domains and share precursors such as acetyl- and malonyl-CoA (Smith and Tsai 2007). The expression profile of genes encoding 3-oxoacyl-(acyl-carrier-protein) synthases (EHK89245.1, EHK87608.1 and EHK84821.1) that play key roles regulating the product distribution of FA synthesis were found to express lower levels in high-primycin producer strain.

Two ABC multidrug transporter encoding genes EHK80158.1 and EHK80159.1 located adjacent to PKS gene cluster (Fig. 4) that are linked to defense mechanisms (V) via COG were overexpressed in *S. azurea* SZMC 14600 with 8.0 and 3.8 log_2_ fold-change respectively. Similarly, TetR family transcriptional regulators (TFRs), involved in control of a variety of processes—such as antibiotic production, efflux pump expression and multidrug and self-resistance—were also overexpressed in high-primycin producer strains within a range of 2.08 to 5.23 log_2_ fold-change.

Although comparative analysis of PBGC did not reveal structural differences between *S. azurea* SZMC 14600 and *S. azurea* DSM 44631, receptor histidine kinase (HK) and a cognate response regulator (RR) encoding genes (Fig. 4), elements of the two components’ signal transduction system (TCS) were significantly up-regulated in high-primycin producer strain. An additional regulatory gene, encoding leucine-responsive regulatory protein (Lrp) was also differentially expressed between the two strains (Table 2).

**Table 2:** Selected up- and down-regulated genes represented by Clusters of Orthologous Groups (COGs) potentially related to primycin biosynthesis in comparison of high- and low-producer *S. azurea* strains, SZMC 14600 and DSM 44631 respectively

COG	Name	Accession no	Relative fold-change (log_2_)	
			*S. azurea* SZMC14600	*S. azurea* DSM 44631
Fatty acid synthesis				
IQ	3-oxoacyl-(acyl-carrier-protein) synthase	EHK89245.1	–	2.07
IQ	3-oxoacyl-(acyl-carrier-protein) synthase	EHK87608.1	–	3.46
I	3-oxoacyl-(acyl-carrier-protein) synthase III	EHK84821.1	–	2.17
Regulation of secondary metabolism				
T	Two-component histidine kinase	EHK80176.1	2.57	–
KT	Two-component system response regulator	EHK80177.1	1.76	–
K	Lrp/AsnC family transcriptional regulator	EHK88701.1	4.24	–
Self-resistance				
V	ABC transporter ATP-binding protein	EHK80158.1	8.01	–
V	ABC-transporter transmembrane protein	EHK80159.1	3.83	–
K	TetR/AcrR family transcriptional regulator	EHK82841.1	2.08	–
K	TetR/AcrR family transcriptional regulator	EHK88308.1	2.33	–
K	TetR/AcrR family transcriptional regulator	EHK82470.1	5.00	–
K	TetR/AcrR family transcriptional regulator	EHK88153.1	5.23	–
K	TetR/AcrR family transcriptional regulator	EHK88592.1	3.16	–
K	TetR/AcrR family transcriptional regulator	EHK83942.1	4.29	–

Abbreviations of COG functional categories: (I) Lipid transport and metabolism; (K) Transcription; (Q) Secondary metabolites biosynthesis, transport and catabolism; (T) Signal transduction mechanisms; (V) Defense mechanisms

## Discussion

There is no doubt that antibiotic resistance is a global and serious issue nowadays (Payne et al. 2015). Even though a number of initiatives have been launched to reinvigorate the antibiotic research and development pipeline, an application of genome mining approach coupled with transcriptomic analysis and analytical methods is not yet common at industry level (Palazzotto and Weber 2018). On the basis of these innovative technologies, this study reports the result of a comparative structural and functional analysis of *S. azurea* strains in terms of primycin producing ability. Reexamination of primycin producing ability among representative species of the genus *Saccharomonospora* revealed that only *S. azurea* strains were capable of synthesizing primycin (Juhász et al. 2011), however the yield of antibiotic was significantly different according to agar well diffusion assay. Comparative HPLC–DAD-ESI/MS analysis of the two primycin producer *S. azurea* strains resulted in remarkable differences not only in yield, but also in dynamics of antibiotic production in each time points of batch fermentation. Enhancement of antibiotic producing ability of industrial strains remains the focus of industry driven research, nevertheless classical strain improvement and optimization of the fermentation process frequently suffers from limitations (Lal et al. 1996; Parekh et al. 2000). In order to assess the similarities and differences between *S*. *azurea* strains in regard to their potential to produce primycin, comparative whole genome analysis was performed. A wide range of biologically active natural products are synthesized by bacterial modular type I PKS assembly line (Hertweck 2015), such as 36-membered marginolactone primycin. Although our hypothesis, that structural differences of PKS gene clusters is responsible for elevated primycin production, was not supported by the comparative in silico analysis. Nevertheless, these efforts revealed the presence of unusual butlymalonyl-CoA, pentlymalonyl-CoA or hexylmalonyl-CoA substrate specificity in module 18. Similar AT domain characteristics were reported in case of stambomycin, thailandi, neoansamycin, antimycin and cinnabaramide biosynthesis (Greule et al. 2016; Li et al. 2015; Rachid et al. 2011; Ray et al. 2016). These findings were further supported by R_2_ side chain variability of primycin molecules (Fig. 5). Regarding quantitative differences in primycin producing ability, much effort has been made in investigating complex gene expression profiles of *S. azurea* strains. RNA-Seq of high- and low-primycin producer *S. azurea* strains resulted 686 DEGs with cutoff 2 and classified into diverse COGs, however metabolism-related COG categories were dominant, representing more than 50% of the total hits (Table 3S). Among them, the agmatinase encoding gene belonging to amino acid transport and metabolism (E) was up-regulated 2.62-fold in the primycin overproducer strain. The relevance of agmatinase gene overexpression highlights its importance in conversion of amino/guanidino marginolactones biosynthesis, as mentioned in an earlier study (Hong 2016). More recently, a case study focused on targeting mechanisms of azalomycin F_5a_ produced by *Streptomyces hygroscopicus* var. *azalomyceticus* revealed that the guanidyl side chain of the marginolactone antibiotic plays pivotal role in antibacterial effect. Consequently, modification of agmatinase enzyme encoding gene expression emerges as a promising target to enhance antibacterial activity (Yuan et al. 2019). Expression profiling revealed DEGs encoding enzymes responsible for fatty acid biosynthesis. The enzymatic machinery responsible for de novo biosynthesis of FAs and polyketides possess many common features, including the utilization of identical precursors (Cronan and Thomas 2009), therefore substrate competition effect theoretically could not be excluded. Our results were based on the actual experimental design, in which high- and low-primycin producer *S. azurea* strains were compared under identical culture conditions, and growth phase did not support the hypothesis that competition generally does not occur due to the time shift of the two biosynthetic process (Gago et al. 2011). The overexpression of FA synthesis related genes in low-producer *S. azurea* DSM 44631 strain suggests that biosynthetic activity of FA and polyketide related machinery is not a clear sequential process, rather should be considered an overlapping even at least partially.

Primycin, a non-polyene marginolactone antibiotic produced by filamentous bacteria *S. azurea*, possesses high antimicrobial activity against frequent G+ pathogens, including clinically prevalent multidrug-resistant strains (Feiszt et al. 2014). To protect themselves against their own bioactive metabolites, self-resistance mechanism for antibiotic producers are crucial. Despite the fact that the history of primycin dates back more than 60 years, there is no available scientific knowledge of the regulation of self-resistance in *S. azurea* species. Among members of the ABC transporter superfamily, encoding genes involved in multidrug and self-resistance (Rees et al. 2009; van Veen and Konings 1998), tightly linked to the PBGC were up-regulated in overproducer strain. Similarly, six genes encoding TetR family of regulators (TFRs), associated with antibiotic resistance (Cuthbertson and Nodwell 2013; Deng et al. 2013), were also significantly overexpressed. These findings support the hypothesis that the expression of resistance genes is presumably induced by primycin or intermediate molecules in a concentration related manner.

A bacterial two component signal transduction system (TCS) is not only important for complex adaptive responses towards environmental changes, but also involved in the biosynthetic control of a broad range of secondary metabolites (Rodríguez et al. 2013; Zschiedrich et al. 2016). For instance the two component system AfsQ1/Q2 of *Streptomyces coelicolor* was found to be capable of stimulating actinorhodin, undecylprodigiosin and calcium-dependent antibiotic production (Wang et al. 2013). Furthermore, it was demonstrated that orf22 and orf23 members of *Streptomyces clavuligerus* TCS positively regulate clavulanic acid biosynthesis, and their overexpression resulted in elevated yield of antibiotic production (Jnawali et al. 2008). To the extent of our knowledge, this is the first report to demonstrate overexpression of HK and RR encoding genes related to elevated primycin biosynthetic capacity in *S. azurea*.

Transcriptional regulation of secondary metabolisms in *Streptomyces* has been extensively studied (Bibb 2005), however there is still room for improvement in case of industrially important rare Actinomycetes. Genes corresponding to Lrp/AsnC family transcriptional regulators were found substantially up-regulated in high-primycin producer strains. Present findings were in agreement with previously reported facts that, genes encoding the Lrp/AsnC family transcriptional regulators (SCO2140 and SCO3361) act as positive regulators of antibiotic production in *S. coelicolor* (Liu et al. 2017; Yu et al. 2016).

In summary, we have presented a comprehensive study based on multidisciplinary approaches e.g. traditional microbiology; analytical chemistry; structural, functional and comparative genomics supported by a wide variety of bioinformatics tools. The primary aim of the research was to gain insight into the difference and determinants of primycin producing ability via comparison of high- and low-antibiotic producer *S. azurea* strains. Even though an in silico analysis of PKS gene clusters did not revealed significant structural differences between the two strains, clear evidence was found for unusual substrate specificity of the AT domain in module 18. Concerning quantitative differences in primycin producing ability, the performed transcriptomic analysis resulted several DEGs, classified into various COG categories. Among them, genes related to fatty acid synthesis, self-resistance, regulation of secondary metabolism and an agmatinase encoding gene responsible for catalyze conversion between guanidino and amino forms of primycin were discussed. As a result our efforts to investigate PBGC and the regulation of primycin biosynthesis provides clues for antibiotic yield- and strain-improvement as well as laying the foundation for rational drug design.

## Electronic supplementary material

Below is the link to the electronic supplementary material.

Supplementary file1 (DOCX 2085 kb)
