# Supplementary material for: Structural and functional comparison of Saccharomonospora azurea strains in terms of primycin producing ability
Source: World J Microbiol Biotechnol. 2020 Sep 29;36(11):160. doi: 10.1007/s11274-020-02935-x (PMC7522111; doi:10.1007/s11274-020-02935-x)
Supplement: Supplementary file 1 — Supplementary file1 (DOCX 2085 kb) [file 11274_2020_2935_MOESM1_ESM.docx]

World Journal of Microbiology and Biotechnology

**Supplementary Materials**

**Structural and functional comparison of *Saccharomonospora azurea* strains in terms of primycin producing ability**

Márk Kovács^1,3^, Dénes Seffer^1^_,_ Ágota Pénzes-Hűvös^1^, Ákos Juhász^2^, Ildikó Kerepesi^3^, Kitti Csepregi^3^, Andrea Kovács-Valasek^3^ and Csaba Fekete^3*^

^1^PannonPharma Pharmaceutical Ltd., H-7720 Pécsvárad, Hungary.

^2^Institute of Biological Sciences, Faculty of Agricultural and Environmental Sciences, Szent István University, H-2100 Gödöllő, Hungary

^3^Institute of Biology, Faculty of Sciences, University of Pécs, H-7624 Pécs, Hungary.

*Corresponding author: E-mail address: fekete@gamma.ttk.pte.hu, Phone: +36 72 503 600

**Table 1S** Comparison of *S. azurea* SZMC 14600 with publicly available genomes of *Saccharomonospora* genus using the Genome-to-Genome Distance Calculator (GGDC)

| **Organism** | **Strain ID.** | **HSP length/total length^1^** | **identities/HSP length^2^** | **identities/total length^3^** |
| --- | --- | --- | --- | --- |
| *S. azurea* | DSM 44631 | 91.30 | 93.40 | 93.90 |
| *S. cyanea* | DSM 44106 | 49.80 | 28.00 | 43.10 |
| *S. glauca* | DSM 43769 | 50.80 | 26.30 | 43.00 |
| *S. xinjiangensis* | DSM 44391 | 41.70 | 24.50 | 36.00 |
| *S. viridis* | DSM 43017 | 33.40 | 22.60 | 29.60 |
| *S. halophila* | DSM 44411 | 18.70 | 22.60 | 18.40 |
| *S. piscinae* | 06168H-1 | 35.70 | 22.30 | 31.00 |
| *S. saliphila* | DSM 45087 | 21.60 | 22.10 | 20.70 |
| *S. paurometabolica* | DSM 44619 | 19.50 | 21.90 | 19.00 |
| *S. marina* | DSM 45390 | 19.30 | 20.30 | 18.70 |

^1^ Length of all HSPs divided by total genome length in %, ^2^ Sum of all identities found in HSPs divided by overall HSP length in %, ^3^ Sum of all identities found in HSPs divided by total genome length in %, where HSP meaning high-scoring segment pairs according to *https://ggdc.dsmz.de*

**Table 2S** Number of protein coding genes with Clusters of Orthologous Groups (COGs) in the five analyzed *Saccharomonospora* genomes

| **Features** | ***S. azurea* SZMC 14600** | | ***S. azurea* DSM 44631** | | ***S. cyanea* DSM 44103** | | ***S. glauca* DSM 43769** | | ***S. viridis* DSM 43017** | |
| --- | --- | --- | --- | --- | --- | --- | --- | --- | --- | --- |
|  | Gene count | % of total | Gene count | % of total | Gene count | % of total | Gene count | % of total | Gene count | % of total |
| Total gene number | 4604 | 100,00 | 4530 | 100,00 | 5196 | 100,00 | 4386 | 100,00 | 3962 | 100,00 |
| Protein coding genes with COGs | 2820 | 61.25 | 2743 | 60.55 | 3228 | 62.12 | 2739 | 62.45 | 2466 | 62.24 |
| Not in COG | 1784 | 38.75 | 1787 | 39.45 | 1968 | 37.88 | 1647 | 37.55 | 1496 | 37.76 |

**Table 3S** Representation of differentially expressed genes of *S. azurea* SZMC 14600 and *S. azurea* DSM 44631 classified into Clusters of Orthologous Groups (COGs)

| **COG function code** | **Functional categories** | **Gene count and % of annotated genes** | |
| --- | --- | --- | --- |
|  |  | **SZMC 14600** | **DSM 44631** |
| ***Information storage and processing*** | | | |
| **J** | Translation, ribosomal structure and biogenesis | 13 (4.4%) | 10 (3.2%) |
| **K** | Transcription | 25 (8.5%) | 20 (6.4%) |
| **L** | Replication, recombination and repair | 10 (3.4%) | 7 (2.2%) |
| ***Cellular processes and signaling*** | | | |
| **D** | Cell cycle control, cell division, chromosome partitioning | 3 (1.0%) | 3 (1.0%) |
| **V** | Defense mechanisms | 12 (4.1%) | 6 (1.9%) |
| **T** | Signal transduction mechanisms | 11 (3.7%) | 10 (3.8%) |
| **M** | Cell wall/membrane/envelope biogenesis | 11 (3.7%) | 22 (7.0%) |
| **N** | Cell motility | 1 (0.3%) | 0 (0%) |
| **W** | Extracellular structures | 0 (0%) | 1 (0.3%) |
| **U** | Intracellular trafficking, secretion, and vesicular transport | 1 (0.3%) | 3 (1.0%) |
| **O** | Posttranslational modification, protein turnover, chaperones | 6 (2.0%) | 10 (3.2%) |
| ***Metabolism*** | | | |
| **C** | Energy production and conversion | 21 (7.1%) | 15 (4.8%) |
| **G** | Carbohydrate transport and metabolism | 17 (5.8%) | 43 (13.7%) |
| **E** | Amino acid transport and metabolism | 33 (11.2%) | 25 (8.0%) |
| **F** | Nucleotide transport and metabolism | 13 (4.4%) | 3 (1.0%) |
| **H** | Coenzyme transport and metabolism | 12 (4.1%) | 19 (6.1%) |
| **I** | Lipid transport and metabolism | 19 (6.5%) | 14 (4.5%) |
| **P** | Inorganic ion transport and metabolism | 20 (6.8%) | 28 (8.9%) |
| **Q** | Secondary metabolites biosynthesis, transport and catabolism | 13 (4.4%) | 15 (4.8%) |
| ***Mobile elements*** | | | |
| **X** | Mobilome: prophages, transposons | 4 (1.4%) | 3 (1.0%) |
| ***Poorly characterized*** | | | |
| **R** | General function prediction only | 39 (13.3%) | 46 (14.7%) |
| **S** | Function unknown | 10 (3.4%) | 10 (3.2%) |
| ∑ COG | | 294 (100%) | 313 (100%) |


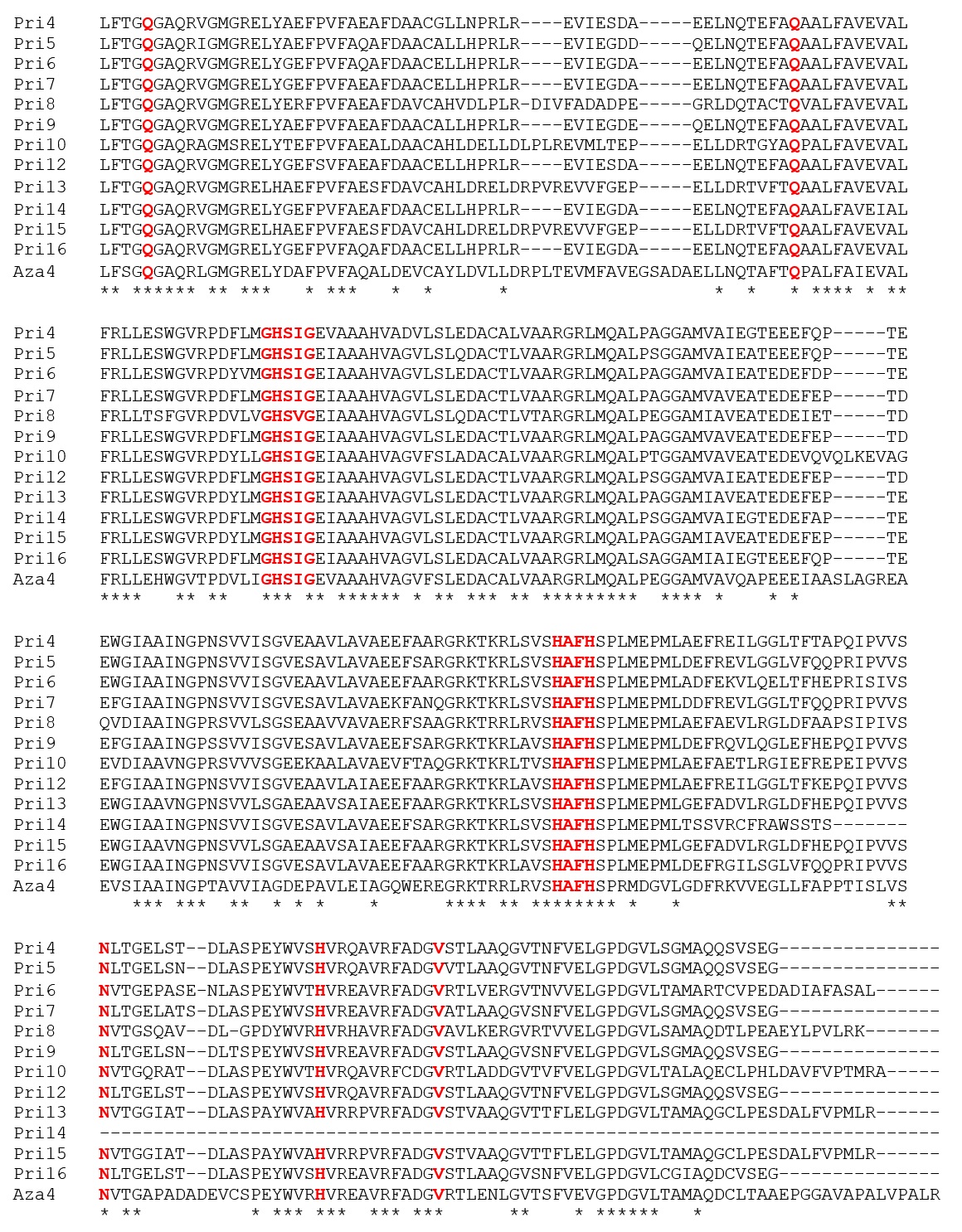


**Fig. 1S** Amino acid sequence alignment of acyltransferases (ATs) with malonyl-CoA substrate specificity in primycin polyketide synthase (PKS). The different AT domain (labelled by Pri) in module 4-10 and 12-16 were aligned with the AT domain (labelled by Aza) of module 4 originated from azalomycin PKS gene cluster of *Streptomyces malaysiensis* DSM 4137. The conserved active site residues of AT domains are highlighted with red, * represents identical amino acids


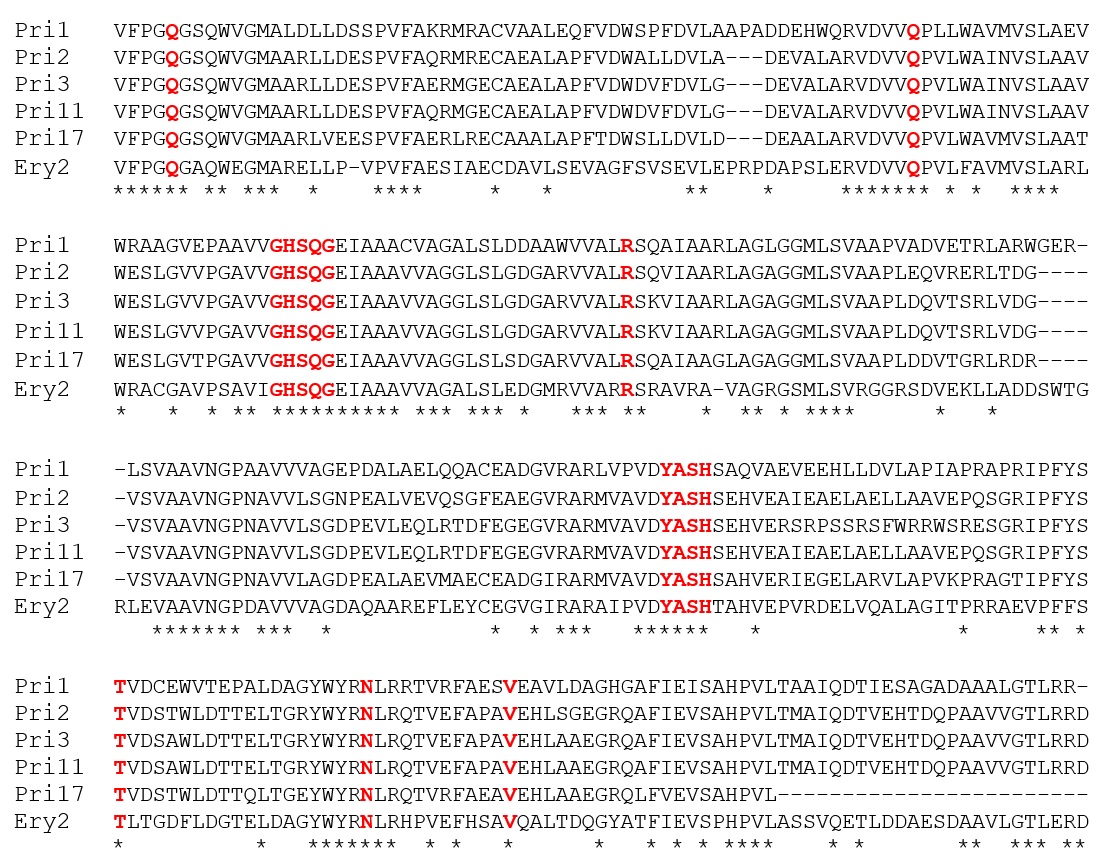


**Fig. 2S** Amino acid sequence alignment of acyltransferases (ATs) with methylmalonyl-CoA substrate specificity in primycin polyketide synthase (PKS). The different AT domain (labelled by Pri) in module 1-3, 11 and 17 were aligned with the AT domain (labelled by Ery) of module 2 originated from erythromycin PKS gene cluster of *Saccharopolyspora erythraea* NRRL 2338. The conserved active site residues of AT domains are highlighted with red, * represents identical amino acids


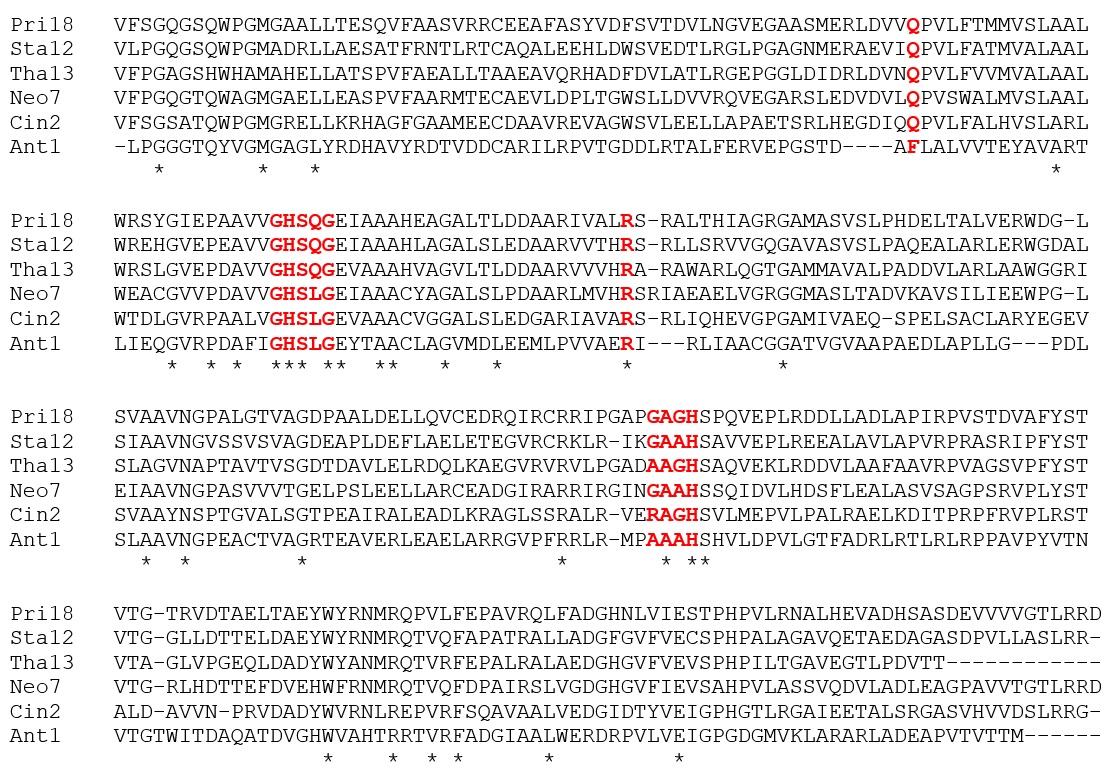


**Fig. 3S** Amino acid sequence alignment of acyltransferase (AT) domains with unusual substrate specificities, represented in type-I polyketide synthase (PKS), responsible for synthesizing different antibiotics. Three letter codes such as Pri, Sta, Tha, Neo, Cin and Ant indicates primycin, stambomycin, thailandin, neoansamycin, cinnabaramide and antimycin acyltransferase (AT) domains, followed by module numbers. Pri18 utilizes butylmalonyl-CoA, pentylmalonyl-CoA and hexylmalonyl-CoA extender units. Sta12 incorporates atypical pentylmalonyl-CoA and hexylmalonyl-CoA extender units. Tha13 can use butylmalonyl-CoA. Neo7 incorporates butylmalonyl-CoA and pentylmalonyl-CoA substrates. Cin2 is specific to hexylmalonyl-CoA substrate and Ant1 shows specificity to propanylmalonyl-CoA, butylmalonyl-CoA, pentylmalonyl-CoA and hexylmalonyl-CoA extender units. The conserved active site residues of AT domains are highlighted with red, * represents identical amino acids


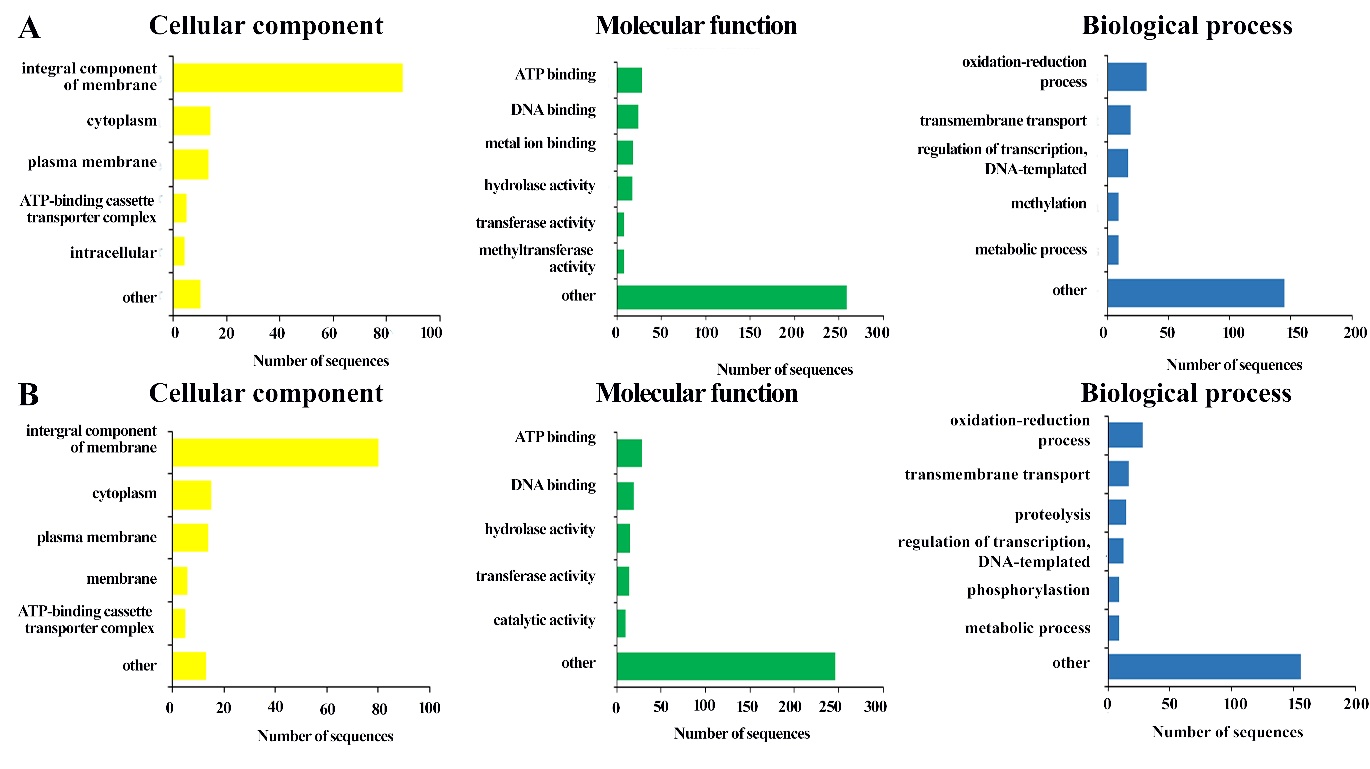
**Fig. 4S** The most abundant Gene Ontology (GO) terms of differentially expressed genes (DEGs) classified into three main GO category. (**A**) *S. azurea* SZMC 14600, (B) *S. azurea* DSM 44631. „Other” indicates representatives of diverse GO terms, where its numbers were less then 5 in the certain GO category


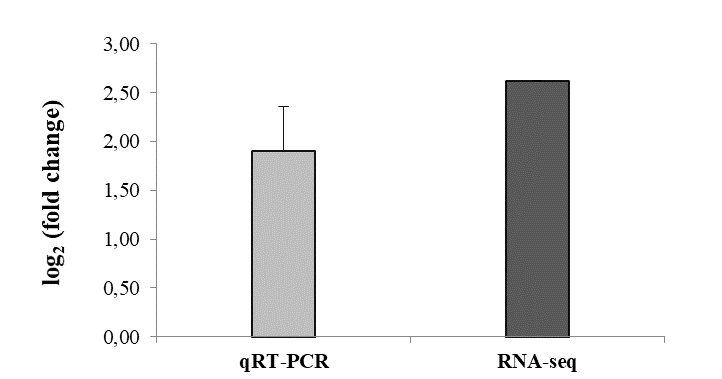


**Fig. 5S** qRT-PCR validation of agmatinase gene expression. The light grey bar represents the mean value of log_2_ transformed fold change of agmatinase gene detected by qRT-PCR, dark grey bar represents RNA-Seq data. Error bar represents standard deviation (does not apply for RNA-Seq data)
